# Artificial intelligence empowering rare diseases: a bibliometric perspective over the last two decades

**DOI:** 10.1186/s13023-024-03352-1

**Published:** 2024-09-13

**Authors:** Peiling Ou, Ru Wen, Linfeng Shi, Jian Wang, Chen Liu

**Affiliations:** 7T Magnetic Resonance Imaging Translational Medical Center, Department of Radiology, Southwest Hospital, Army Medical University, (Third Military Medical University), 30 Gao Tan Yan St, Chongqing, 400038 China

**Keywords:** Rare diseases, Artificial intelligence, Bibliometric analysis, Medical informatics

## Abstract

**Objective:**

To conduct a comprehensive bibliometric analysis of the application of artificial intelligence (AI) in Rare diseases (RDs), with a focus on analyzing publication output, identifying leading contributors by country, assessing the extent of international collaboration, tracking the emergence of research hotspots, and detecting trends through keyword bursts.

**Methods:**

In this bibliometric study, we identified and retrieved publications on AI applications in RDs spanning 2003 to 2023 from the Web of Science (WoS). We conducted a global research landscape analysis and utilized CiteSpace to perform keyword clustering and burst detection in this field.

**Results:**

A total of 1501 publications were included in this study. The evolution of AI applications in RDs progressed through three stages: the start-up period (2003–2010), the steady development period (2011–2018), and the accelerated growth period (2019–2023), reflecting this field’s increasing importance and impact at the time of the study. These studies originated from 85 countries, with the United States as the leading contributor. “Mutation”, “Diagnosis”, and “Management” were the top three keywords with high frequency. Keyword clustering analysis identified gene identification, effective management, and personalized treatment as three primary research areas of AI applications in RDs. Furthermore, the keyword burst detection indicated a growing interest in the areas of “biomarker”, “predictive model”, and “data mining”, highlighting their potential to shape future research directions.

**Conclusions:**

Over two decades, research on the AI applications in RDs has made remarkable progress and shown promising results in the development. Advancing international transboundary cooperation is essential moving forward. Utilizing AI will play a more crucial role across the spectrum of RDs management, encompassing rapid diagnosis, personalized treatment, drug development, data integration and sharing, and continuous monitoring and care.

**Supplementary Information:**

The online version contains supplementary material available at 10.1186/s13023-024-03352-1.

## Introduction

Rare diseases (RDs), also known as orphan diseases, are generally defined by an incidence rate of less than 1 in 2,000 individuals [1]. Globally, over 7,000 types of RDs are recognized, impacting more than 350 million individuals, nearly 50% of whom are children [2]. Although each rare disease (RD) may only affect a relatively small number of individuals, RDs are not rare as a group, imposing significant health and economic burdens on patients and society [3]. The uniqueness of RDs poses challenges to traditional diagnostic and treatment paradigms [4]. The complexity and variability of these diseases tend to result in low recognition rates, high misdiagnosis rates, and delayed diagnoses [5]. Given the genetic basis and early onset of approximately 80% of RDs, along with their potential for progressive severe disabilities or death [6], there is an urgent need for innovative diagnostics and advanced technologies to improve the overall management of these conditions.

Despite these challenges, advances in medical research and technology, particularly in the field of AI, have shown great promise in transforming healthcare, including the diagnosis, treatment, and management of RDs [7]. AI, a branch of computer science that creates intelligent machines capable of tasks such as natural language understanding and complex problem-solving [8], assists healthcare professionals by analyzing vast medical data to inform accurate diagnoses and treatment decisions [9]. In recent years, scholars have increasingly focused on applying AI to RDs, recognizing its potential to reveal complex patterns [10]. A comprehensive understanding of this field can help identify research priorities, guide future directions, and improve clinical practice.

Bibliometric analysis has emerged as a pivotal research method, utilizing mathematical and statistical techniques to quantitatively assess and reveal the trends and evolution of research hotspots [11]. As the volume of scientific literature continues to expand and the significance of evaluating research impact grows, bibliometrics plays a crucial role in identifying emerging trends and potential areas for future research [12]. Scholars have applied this method to analyze the application of AI in various diseases, including studies on liver fibrosis [13], diabetic retinopathy [14], and Covid-19 [15]. To our knowledge, there has yet to be a systematic bibliometric analysis of AI applications in RDs. Therefore, this study will fill the gap in the existing literature by synthesizing the current findings to provide a comprehensive overview in this field.

## Materials and methods

### Study design

This cross-sectional study conducted a bibliometric analysis of publications on AI applications in RDs over the last two decades and followed the Strengthening the Reporting of Observational Studies in Epidemiology (STROBE) guideline. Since all data were obtained directly from the database, with full records available for analysis, no ethical review was necessary.

### Data acquisition and search strategy

**Fig. 1:**
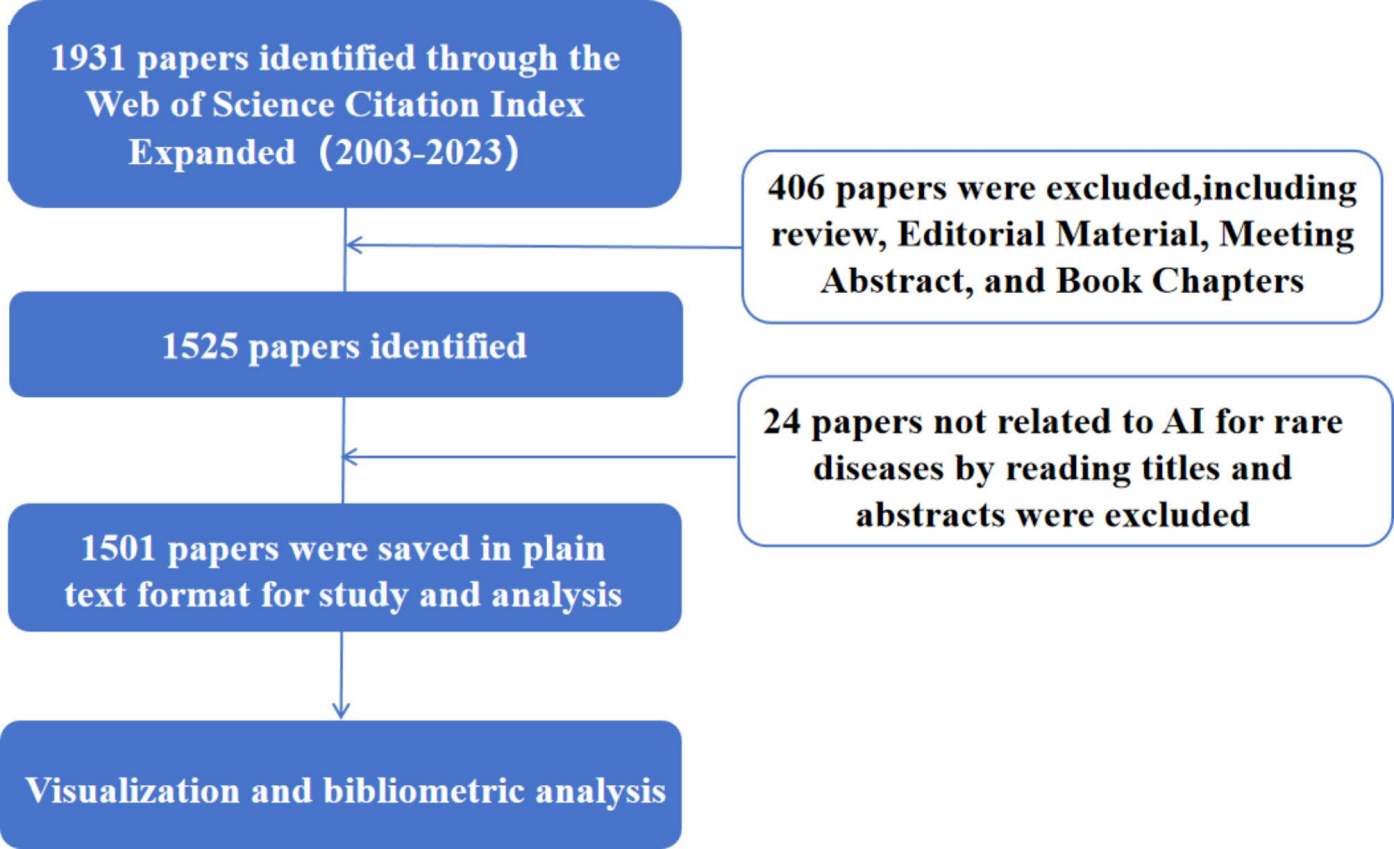
Flow-chart of this study

In this study, we employed a strategy of formulating a search query to retrieve original data from the Web of Science (WoS). The WoS database, as a widely recognized comprehensive academic database, provides interdisciplinary literature resources, detailed citation information, and timely data updates, making it a comprehensive and reliable source for our analysis [16, 17]. Two senior clinical professors (Dr. Liu and Dr. Wang) jointly discussed and determined the search strategy, which were reviewed by the professional librarian (Mrs. Zhao). We identified literature on AI applications in RDs using the following search terms: TS = (“AI” OR “artificial intelligen*” OR “data learning” OR “robotic*” OR “computer vision” OR “machine learning” OR “deep learning” OR “deep network*” OR “neural learning” OR automat* OR algorithm OR “neural network*” OR “expert* system*”) AND TS=(“rare disease*” OR “rare disorder*” OR “orphan disease*” OR “infrequent disease*” OR “seldom disease*” OR “ultra-rare disease*” OR “orphan medicinal*” OR “rare” NEAR/5 “disease*” OR “rare” NEAR/5 “disorder*” OR “orphan*” NEAR/5 “disease*”) AND DOP*= (2003-01-01/2023-12-31). We also compared the total number of publications in the medical field during the same period. This comparison enables us to evaluate if the trend of AI application in RDs aligns with the overall trend in medical AI research. Initially, a total of 1,931 papers within the field of AI applications in RDs were identified through our search terms. To refine our analysis, only articles were included, excluding other types of documents including reviews (345), editorial material (28), meeting abstracts (28), and book Chaps. (5). Then, two researchers (Dr. Ou and Dr. Shi), carefully reviewed the titles and abstracts of each publication, excluding 24 articles that were not related to AI applications in RDs. Complete records were then extracted from relevant publications, saved in plain text format for further research. We listed the details of these publications in the supplementary material. The detailed process of literature search and screening was shown in Fig. 1.

### Data analysis

In our global research landscape analysis, we created a map to visualize the geographical distribution of publications on AI applications in RDs. The geographical map used varying shades of color to represent differences in publication volume, effectively showcasing the research output across various countries worldwide. Additionally, we constructed international cooperation networks to highlight the collaborative relationships among the 10 most productive countries.

To further understand the patterns and trends within the literature, we employed CiteSpace software (Version 6.1.R6) to generate co-occurrence networks of keywords. This approach helped us identify research hotspots and create a visual knowledge map, effectively translating complex information into accessible and appealing presentations [18]. Keywords served as a high-level summary of an article, and the co-occurrence analysis of keywords made it possible to reveal relevant research hotspots [19]. Keywords clustering and burst detection were utilized to detect research hotspots and track the evolution of scientific discourse [20]. Specifically, burst detection was developed for capturing significant increases in keyword popularity within a set timeframe [21]. These techniques allowed for the efficient categorization and sorting of complex data, simplifying the classification and analysis of research findings [22]. In a visual network graph, a larger node size suggested a greater number of publications within a given research area [23]. The Logarithmic Likelihood Ratio (LLR) algorithm was deployed to explore clusters and extract important phrases [11]. The *Q* value (Modularity) and *S* value (Silhouette) were two primary parameters for evaluating cluster quality. Generally, a *Q* value greater than 0.3 indicates significant modularity within clusters, while an *S* value above 0.5 suggests a reasonable clustering [24]. Nodes displayed with purple rings represented those with high betweenness centrality, which tend to play a significant role in the development of the scientific field [25]. These metrics allowed us to create a graphical representation that reveals the research status and trends within complex textual data.

## Results

### Annual trend of publications

**Fig. 2:**
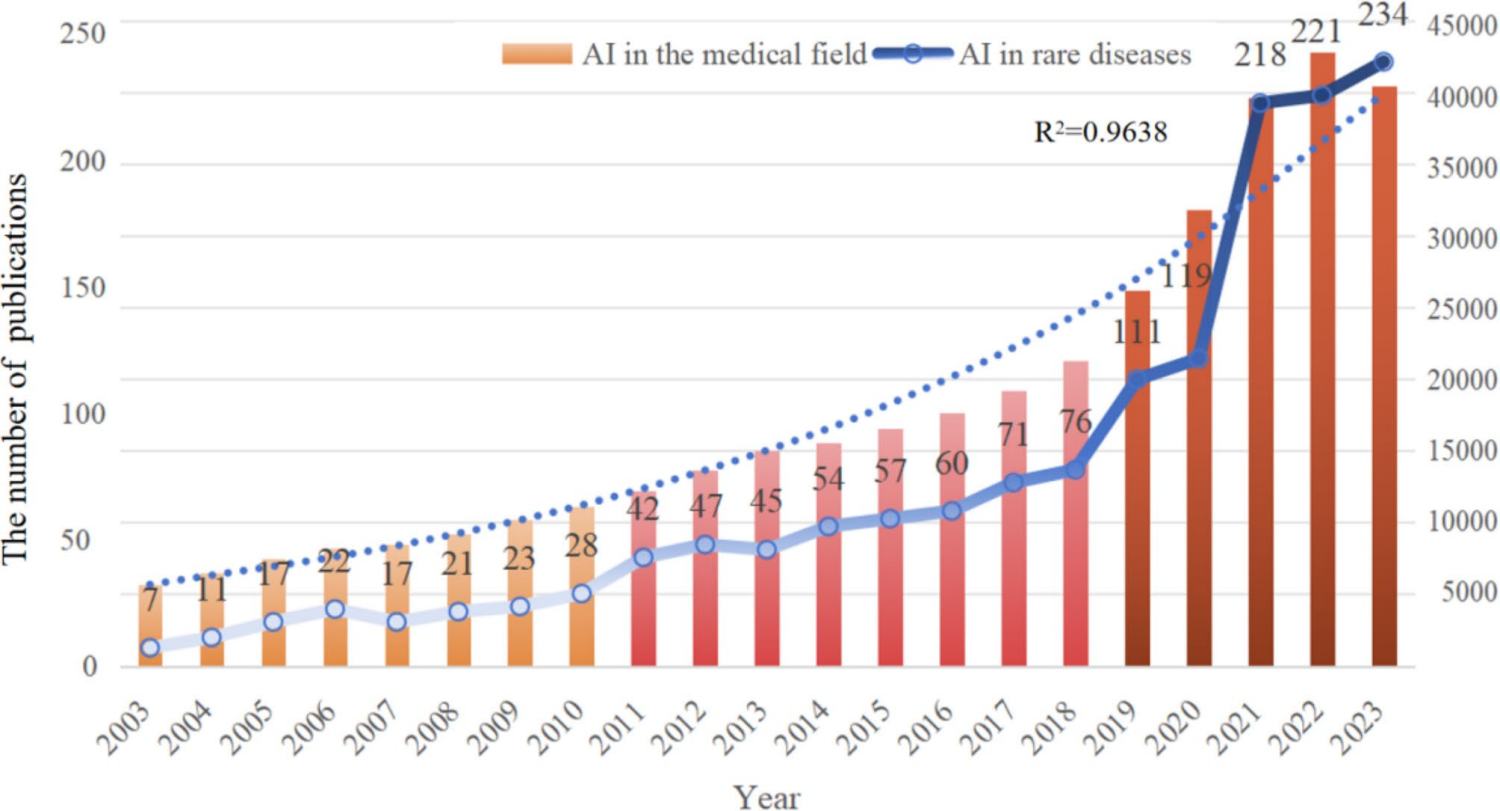
Annual output of publications on artificial intelligence applications in rare diseases and the overall medical field from 2003 to 2023

From 2003 to 2023, 1501 articles were published in the field of AI applications in RDs, averaging 75 papers annually. Figure 2 illustrates the number of publications related to AI in RDs in comparison to the total number of publications in the medical field. The evolution of this field can be divided into three stages: the start-up period (2003–2010), the steady development period (2011–2018), and the accelerated growth period (2019–2023). During the start-up period, no more than 30 articles were published annually, with the number of publications gradually increasing from 1 to 28. In the second stage, this field entered a stable phase, with the average annual number of publications increasing from 28 to 56. Notably, the year 2021 marked a turning point, as the number of publications surged from 119 in 2020 to 218 in 2021, demonstrating an 83.19% increase in the growth rate. This field then entered an accelerated phase of development, with the average annual number of publications nearly doubling from the previous period. To quantify this trend, the exponential growth function was used to evaluate the relationship between cumulative publications and publication year, showing a strong correlation with the trend in the cumulative number of publications (*R*^2^ = 0.963).

### Distribution of countries/regions in the field of AI applications in RDs

**Fig. 3:**
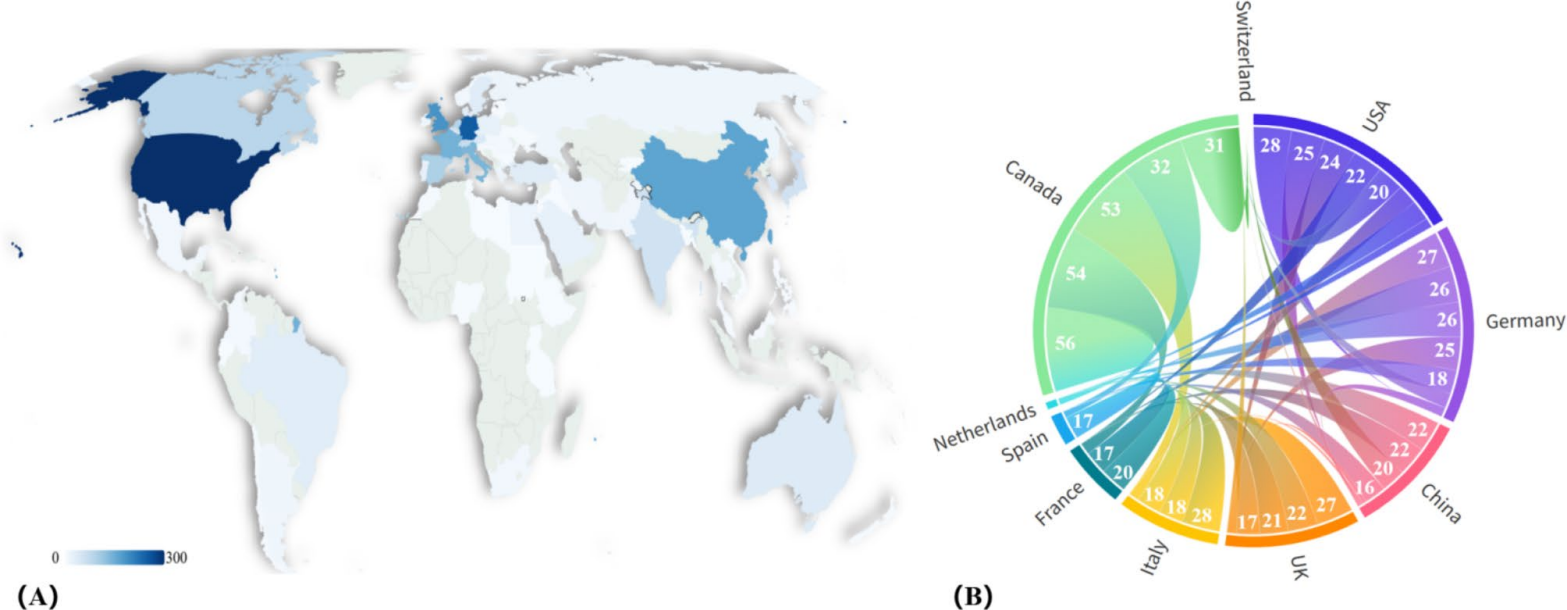
The global landscape of artificial intelligence applications in rare diseases (**A**) Geographical distribution of articles (**B**) The cooperation graph of 10 highly productive countries

We depicted the geographical distribution of AI publications in RDs in Fig. 3(A), which covered outputs from over 85 countries/regions. The United States had the highest number of publications in the field of AI applications in RDs, with 515 publications accounting for 35.23% of the total. This was followed by Germany with 235 publications (16.07%), England with 172 publications (11.77%), China with 152 publications (10.40%), and France with 135 publications (9.23%). Figure 3(B) showed the cooperation relationships among the top 10 countries with the highest collaboration rates, highlighting the ‘’bridging’’ role played by Canada, the United States, and Germany in this field.

### Keyword co-occurrence analysis in the field of AI applications in RDs

**Fig. 4:**
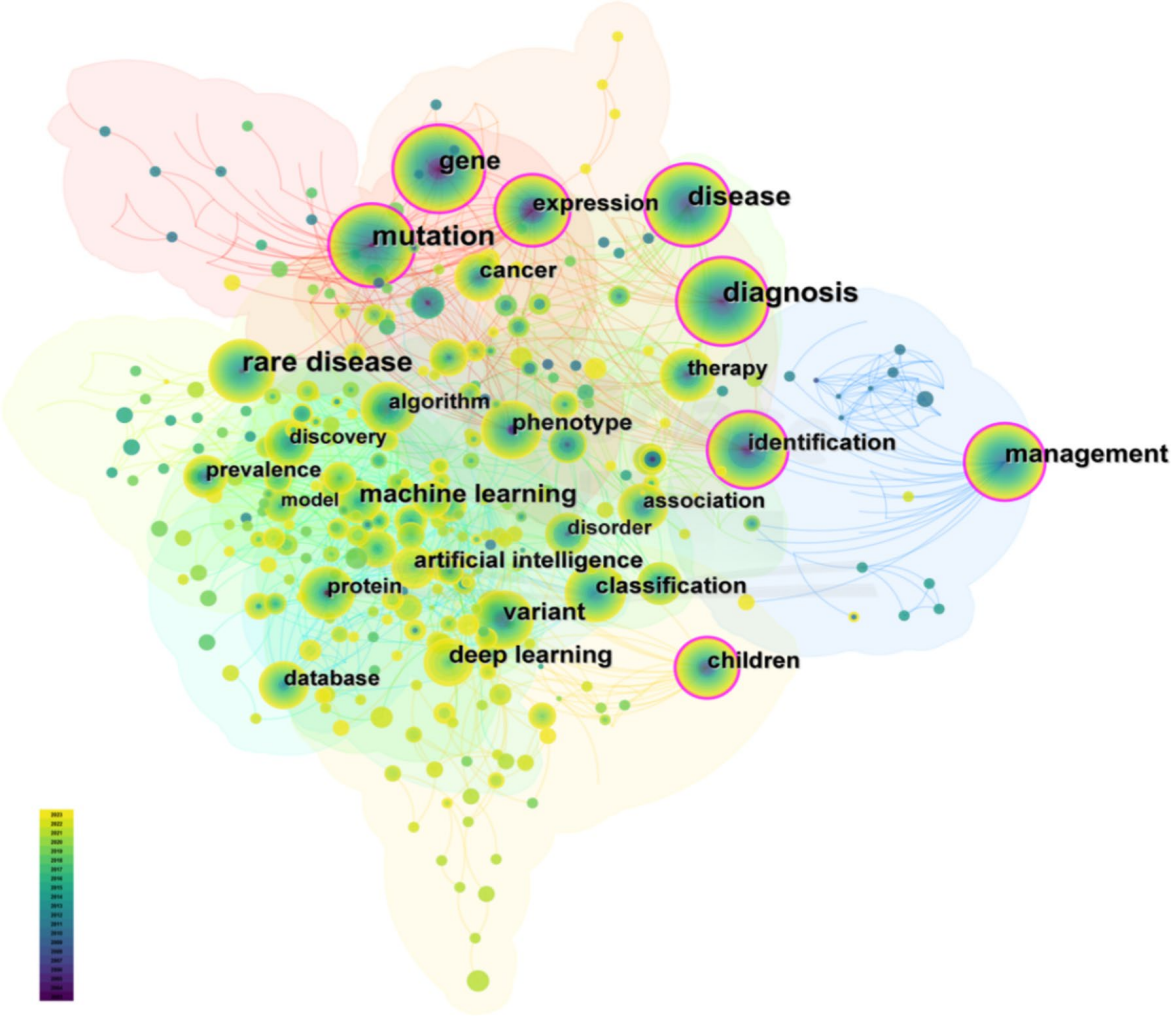
Keyword co-occurrence network map (*Q* = 0.5895, *S* = 0.8061)

To enhance our analysis, we have excluded any keywords that are identical to the search terms. “Mutation”, “Diagnosis” and “Management” were the top 3 keywords with both high frequency and high betweenness centrality. Table 1 lists the top 20 keywords with high frequency and high betweenness centrality. We constructed a keyword co-occurrence map using CiteSpace (Fig. 4), which consists of 628 nodes and 1630 edges. This map reveals a broad spectrum of research topics related to AI applications in RDs, as well as the close interconnections between these topics.

### The evolution of the research hotspots: keyword clustering analysis

To better understand the development patterns of AI in the field of RDs, we divided the publications into three distinct stages based on publication trends over time, creating keyword clustering maps for each: (i) the start-up period (2003–2010), (ii) the steady development period (2011–2018), and (iii) the accelerated growth period (2019–2023).

**Fig. 5:**
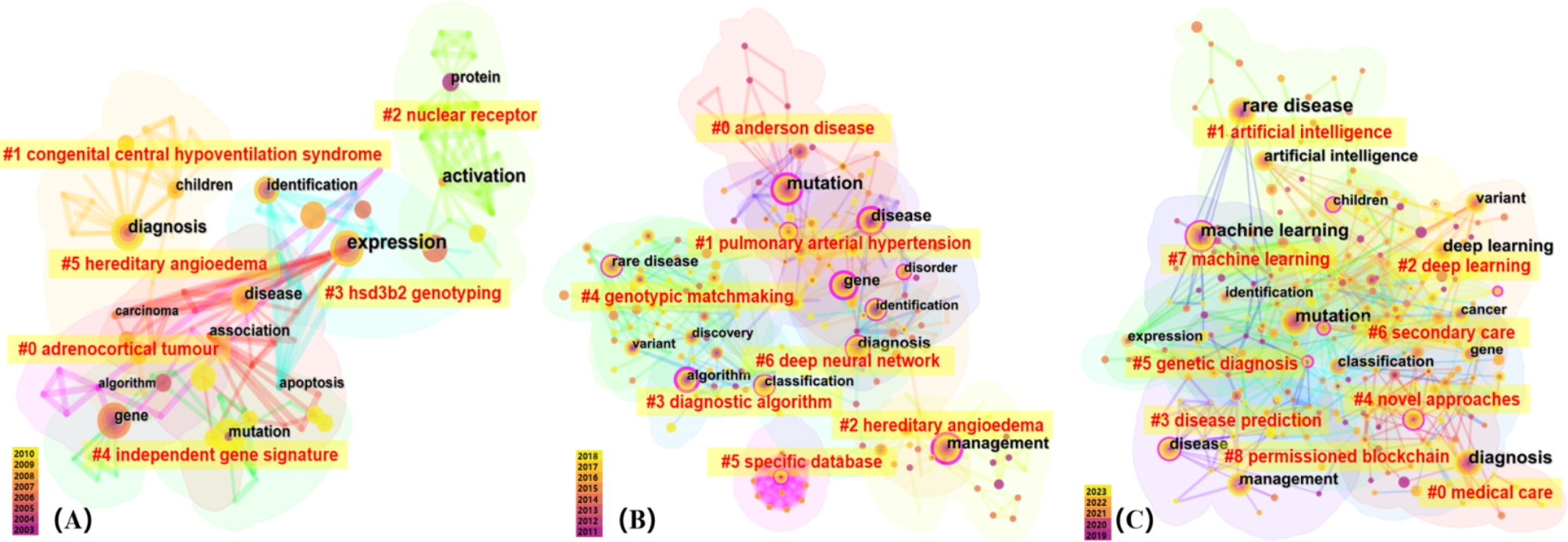
The knowledge map of keyword clustering. (**A**) 2003–2010 (*Q* = 0.5935, *S* = 0.7807) (**B**) 2011–2018 (*Q* = 0.5701, *S* = 0.8203) (**C**) 2019–2023 (*Q* = 0.5895, *S* = 0.8061)

Figure 5(A) displays that the primary clusters formed during the start-up period are as follows: Cluster #0 Adrenocortical tumor, #1 Congenital central hypoventilation syndrome, and #2 Nuclear receptor. These clustering results in the start-up period reflect the preliminary exploration of specific diseases and potential targets, laying the groundwork for future research.

Figure 5(B) illustrates the keyword clustering during the steady development period from 2011 to 2018. It is noteworthy that during this time, we observe the gradual emergence of AI-related clusters, such as #3 diagnostic algorithm, #5 specific database, and #6 deep neural network. These clustering results indicate that researchers have been exploring the application of AI in the field of RDs, with a focus on developing and optimizing diagnostic algorithms, constructing and utilizing specialized databases, and applying deep learning techniques.

Figure 5(C) demonstrates that from 2019 to 2023, an increasing number of scholars have focused on this field, leading to the emergence of more diverse clusters. The prominent clusters for research have transformed into: #0 Medical Care, #1 Artificial Intelligence, #2 Deep Learning, and #3 Disease Prediction. This indicates that scholars have paid more attention to the application of AI to improve healthcare delivery and patient outcomes with RDs, ranging from various healthcare applications to advanced data analysis techniques and disease prediction.

### Future research trends of AI applications in RDs: keyword burst detection

**Fig. 6:**
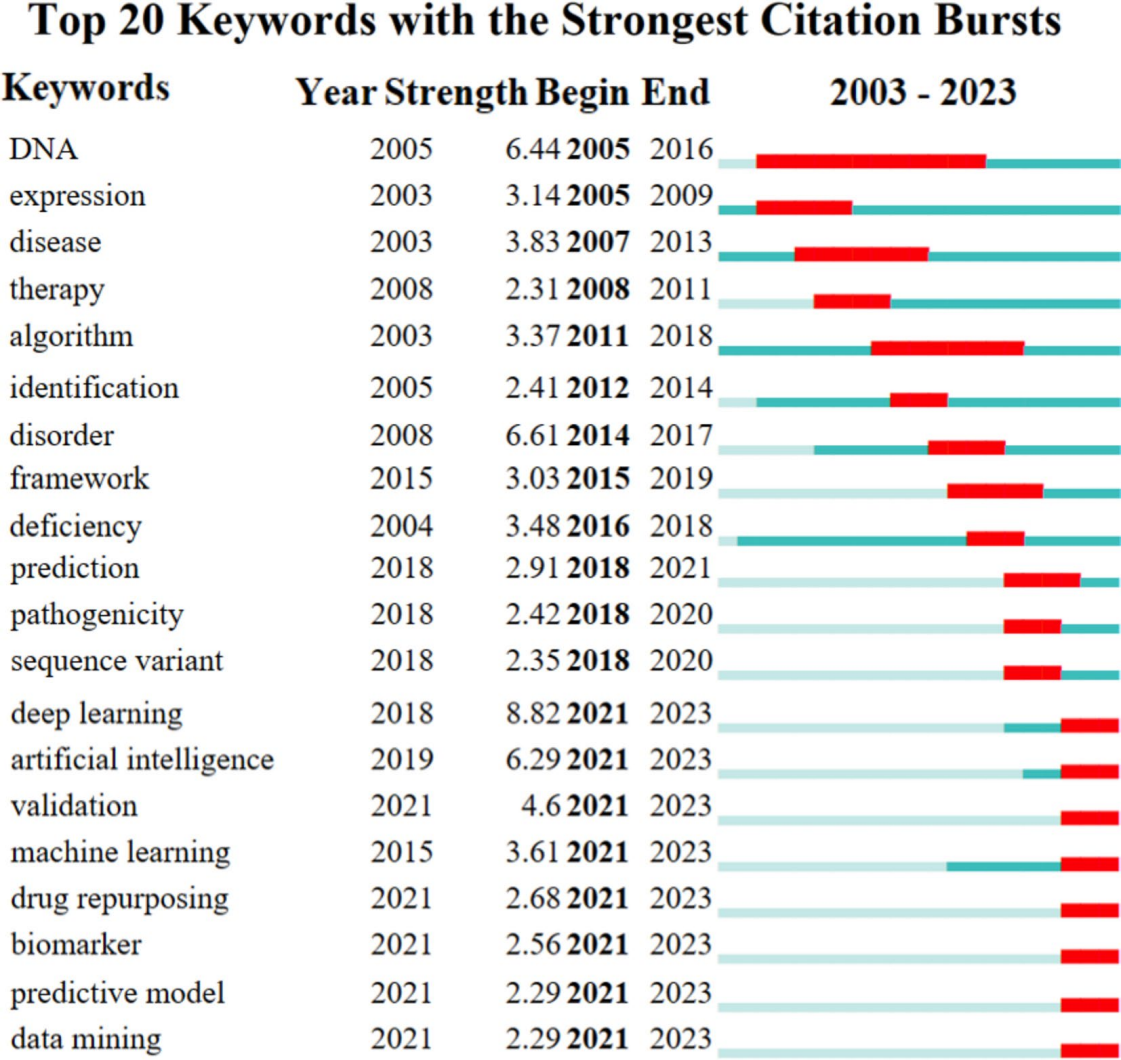
Top 20 keywords with the strongest citation bursts

Furthermore, CiteSpace was utilized to detect keyword bursts by its built-in algorithm to map out the evolving research frontiers within this field. Figure 6 presents the top 20 keywords with the strongest citation bursts from 2003 to 2023. ‘’DNA’’ showed the longest burst duration, spanning 11 years. Along with ‘’disorder’’ and ‘’deep learning’’, it was one of the top 3 keywords with the strongest citation bursts, each with a burst strength of more than 6.0. The recent focus on “biomarker,” “predictive model,” and “data mining” in research highlighted the growing interest in these areas and their potential to shape future research directions.

## Discussion

Our study utilized a bibliometric approach to explore the application of AI in RD research over the past 20 years, focusing on analyzing publication output, identifying leading countries, assessing international collaboration, tracking the emergence of research hotspots, and detecting trends in keyword bursts.

The annual trend of publications results indicate a gradual increase in publications on AI applications in RDs from 2003 to 2023, with accelerated growth particularly notable in the past five years. The trend of AI applications in RDs is consistent with the broader trends in medical AI research, indicating that RD research is not only keeping pace with general medical studies but also might benefit from the extensive application and innovation of AI technology in healthcare [26]. The exponential growth in publication trends indicates both a heightened awareness within the scientific community and the increasingly significant role of AI in empowering research related to RDs [27]. The complex and intricate conditions of RDs require innovative solutions supported by quantitative and specialized tools to assist in the decision-making process [28]. AI algorithms, with their ability to rapidly and comprehensively analyze vast amounts of genetic data [29], have bolstered the identification of RDs-related patterns and biomarkers, improving diagnostics and personalizing treatment strategies [30, 31]. Furthermore, the emergence of Large Language Models (LLMs) has demonstrated significant potential in the field of RDs by enabling advanced human-computer interactions, facilitating the analysis and optimization of extensive data, and enhancing capabilities in diagnosing, risk-predicting, and medical management [32]. Building on these advances, the ongoing exploration and development of AI applications in RD research are opening up a promising horizon for scientific discovery and therapeutic advancement.

The geographical distribution and collaborative patterns analysis not only highlights the leading countries in AI applications for RD research but also emphasizes the value of a collaborative global approach. The United States emerges as the most productive country, accounting for 35.23% of the total publications. The high productivity of the United States can be attributed to its leading development in data science, and the country-specific regulatory frameworks [33, 34]. Collaborative networks further illustrate the critical role played by the United States, Canada, and Germany in fostering international cooperation. Given the complex etiology and pronounced geographical variability of RDs, academic collaboration, while inherently challenging, is indispensable for driving research forward [35]. The integration of multinational and multidisciplinary expertise, as demonstrated by numerous pre-clinical and clinical initiatives, yields novel insights into RDs [36]. International collaboration is thus vital for advancing AI-driven solutions in RD research, laying the groundwork for innovation and progress, and equipping us to tackle the unique challenges posed by these conditions more effectively.

Keyword co-occurrence analysis offers a comprehensive visualization of research topics related to AI applications in RDs. High-frequency keywords highlight current research priorities, including understanding genetic mutations, improving diagnostic techniques, and developing personalized management plans. In genetic diagnosis, AI advancements in statistical methods and deep learning are revolutionizing the detection, interpretation, and prediction of pathogenic splicing variants through RNA-seq data and long-read sequencing technologies [37, 38]. For RDs management, AI-powered tools enable continuous patient data monitoring [39], aiding drug development and improving outcomes [40]. Keyword burst detection reveals current trends in personalized and data-driven healthcare, emphasizing the potential of innovative technologies to enhance RD diagnosis and treatment. The pipeline of potential orphan products grows significantly with AI’s contributions to clinical trial design and data analysis, promising pioneering interventions in RD treatments [41]. Moreover, the integration of multiomics analysis is a focal point for future breakthroughs in RDs, necessitating more powerful algorithm updates and funding support [42, 43].

Our study innovatively explored the evolution of research hotspots of AI applications in RDs across three distinct stages. The clustering outcomes elucidate a paradigm shift in the field of AI application in RDs, evolving from nascent exploration to an era marked by extensive utilization and sophisticated technological amalgamation. Initially, scholarly efforts were predominantly focused on identifying and elucidating the fundamental pathology of specific diseases, indicating the initial exploration of disease mechanisms [44]. With the advancement of this field, the emergence of AI-related clusters mirrored the ascendancy of personalized therapeutic approaches for RDs, as well as the integration of novel technological innovations [45]. Machine learning serves as a valuable tool for extracting disease-relevant patterns from these high-dimensional datasets, offering insights into genes, molecular pathways, and cell types associated with disease phenotypes [46]. Furthermore, in response to the increasing demand for strategies to manage extensive biomedical data, AI plays a crucial role in addressing individual variations and refining pertinent diagnostic assays, thereby offering more precise treatment plans for patients with RDs [47]. Looking ahead, the trajectory of AI applications in RDs suggests a future where personalized medicine becomes increasingly accessible [48]. With AI’s ability to analyze vast amounts of data and tailor treatments to individual patient profiles, we can expect significant strides in precision medicine for rare diseases. This will not only improve patient outcomes but also reduce the time and cost associated with traditional drug development and treatment paradigms.

There are some limitations in this study. We utilized the WoS database for literature search, which may not provide a comprehensive representation of all published literature. Meanwhile, due to the time lag between publication and inclusion in the database, some recently published articles may not have been included. However, the extensive scope and authoritative status of the WoS database substantiate the reliability and significance of our study results [49].

## Conclusion

To our knowledge, this is the first comprehensive analysis to revealing the progress and dynamics of research in the field of AI applications in RDs. Our results show that AI has been widely applied in RDs and is experiencing accelerated growth. International transboundary cooperation among countries/regions should be strengthened in the future. Future trends are expected to prioritize the application of advanced technologies and algorithmic approaches to enhance identification and management of RDs, and to facilitate better utilization of AI algorithms for improved diagnosis and treatment outcomes. Through continuous innovation and the integration of AI technologies, more precise and efficient diagnostic and treatment solutions can be provided for RD patients, bringing new hope to the research and clinical practice in the field of RDs.

**Table 1:** The top 20 high-frequency keywords of artificial intelligence applications in rare diseases

Rank	Keywords	Count	Centrality	Rank	Keywords	Count	Centrality
1	Mutation	146	0.21	11	Phenotype	43	0.05
2	Diagnosis	126	0.16	12	Algorithm	41	0.07
3	Management	88	0.19	13	Association	41	0.04
4	Gene	73	0.12	14	Therapy	36	0.04
5	Variant	55	0.08	15	Protein	36	0.06
6	Identification	53	0.09	16	Database	34	0.03
7	Classification	53	0.08	17	Prevalence	34	0.03
8	Children	52	0.07	18	Discovery	32	0.04
9	Expression	49	0.08	19	Disorder	29	0.05
10	Cancer	46	0.07	20	Model	23	0.01

## Electronic supplementary material

Below is the link to the electronic supplementary material.

Supplementary Material 1

## Data Availability

The datasets generated and analysed during the current study are available in the Web of Science database, https://webofscience.clarivate.cn/wos/woscc/basic-search.

## Abbreviations

RDs: Rare diseases
RD: Rare disease
AI: Artificial intelligence
WoS: Web of Science
LLMs: Large Language Models
